# Non-Orthogonality Measure for a Collection of Pure Quantum States

**DOI:** 10.3390/e24050581

**Published:** 2022-04-21

**Authors:** Kentaro Kato

**Affiliations:** Quantum ICT Research Institute, Tamagawa University, 6-1-1 Tamagawagakuen, Machida 194-8610, Tokyo, Japan; kkatop@lab.tamagawa.ac.jp

**Keywords:** quantum communications, quantum cryptography, quantum states, non-orthogonality, least squares error, *M*-ary optical signal

## Abstract

Modern optical communication technology can realize a large-scale multilevel (or *M*-ary) optical signal. Investigating the quantum mechanical nature of such a large-scale *M*-ary optical signal is essential for a unified understanding of quantum information science and optical communication technology. This article focuses on the quantum-mechanical non-orthogonality for a collection of pure quantum states and proposes a non-orthogonality index based on the least squares error criterion in quantum detection theory. First, we define the index for linearly independent signals, and the proposed index is analyzed through numerical simulations. Next, the index is applied to a highly large-scale *M*-ary phase-shift keying (PSK) coherent state signal. Furthermore, the index is compared with the capacity of the pure state channel with the PSK signal. As a result, it is shown that a highly large-scale *M*-ary PSK coherent state signal exhibits a quantum nature even when the signal transmission power is very high. Thus, the theoretical characterization of a highly large-scale *M*-ary coherent state signal based on the proposed index will be the first step toward a better understanding of cutting-edge optical communication technologies such as the quantum stream cipher Y00.

## 1. Introduction

In 1967–1968, Helstrom achieved a breakthrough in optical communication theory by providing a new framework with a complete quantum mechanical description of optical signals and receivers [1,2,3]. In addition, he successfully demonstrated the quantum limit of detection error for binary optical signals based on the Bayes and Neyman–Pearson criteria developed in the classical detection theory (e.g., [4,5]). After Helstrom’s work, Yuen et al. investigated the conditions for the optimal quantum detection of general quantum states based on a linear programming method [6,7]. Furthermore, Holevo investigated the existence problem for optimal quantum detection and demonstrated the necessary and sufficient conditions for the optimal quantum detection of general quantum states [8]. These pioneering scientists opened up the field of quantum detection theory. Quantum detection theory has since been extensively developed and is a key theory for unifying quantum information science and optical communication technology.

In quantum detection theory, optical signals are mathematically expressed as quantum states of light. For pure states, error-free quantum detection is only allowed when the states are orthogonal to each other. This is a significant result of quantum detection theory. A similar result is observed from the no-cloning theorem [9,10,11]. The no-cloning theorem claims that perfect cloning is possible within a collection of quantum states if and only if the quantum states are orthogonal.

Recent development in experimental studies on the quantum stream cipher Y00 demonstrates that highly large-scale multilevel (or *M*-ary [12,13,14]) optical signals can be realized using advanced technologies in optical communications [15,16]. Therefore, the theoretical characterization of a large-scale collection of coherent states is essential for a unified understanding of quantum information science and optical communication technology.

Coherent states are non-orthogonal, and a collection of coherent states forms a linearly independent set. Hence, the case of linearly independent pure states is of particular interest. A collection of pure states can be almost orthogonal, moderately non-orthogonal, or almost identical states. Therefore, a quantitative measure of the degree of non-orthogonality of each collection is needed for a detailed analysis. In the case of binary pure states, the degree of non-orthogonality is usually measured through the modulus of the inner product between the two states. However, no method to quantify the degree of non-orthogonality of a collection of more than three quantum states has been developed. Therefore, this study aims to develop a quantitative measure for the non-orthogonality of a collection of many states.

For this aim, we propose an index to evaluate the non-orthogonality of a collection of linearly independent pure states based on the least squares error (LSE) criterion in quantum detection theory. We summarize the LSE criterion in Section 2 and define a non-orthogonality index in Section 3. The proposed index is analyzed through numerical simulations with randomly generated vectors in Section 4. Then, the index is applied to the *M*-ary phase-shift keying (PSK) coherent state signal in Section 5. Further, the capacity of a pure state channel with the PSK signal is analyzed to understand the operational meaning of the index in the same section. Finally, we give conclusions in Section 6.

## 2. LSE Criterion in Quantum Detection Theory

Let $S=\{|\psi_{m}\rangle :1\leq m\leq M\}$ be a collection of *M* linearly independent pure quantum states, where each state is normalized, $∥\psi_{m}∥=1$. Then, the squared error E(S,β) for *S* by adapting an orthonormal basis $\beta =\{|v_{m}\rangle :1\leq m\leq M\}$ in vector space V spanned by *S* as a measurement basis is defined as follows.

(1) $$E\left(S,\beta \right)=\frac{1}{M}\sum_{m=1}^{M}\langle e_{m}|e_{m}\rangle ,$$

where $|e_{m}\rangle =|\psi_{m}\rangle -|v_{m}\rangle$. This expression can be arranged into the following form:

(2) $$E\left(S,\beta \right)=\frac{1}{M}\sum_{m=1}^{M}∥e_{m}{∥}^{2}=\frac{1}{M}\sum_{m=1}^{M}{∥\psi_{m}-v_{m}∥}^{2}.$$

Then, the least squares error (LSE) is defined as

(3) $$E^{\circ }\left(S\right)=\min_{\beta }E\left(S,\beta \right)=E\left(S,\beta^{\circ }\right).$$

A constructive manner can find the optimal basis $\beta^{\circ }$ from past studies as follows.

**Theorem** **1** ([17,18]). *For $S=\{|\psi_{m}\rangle :1\leq m\leq M\}$ of linearly independent pure quantum states, the optimal basis $\beta^{\circ }=\{|v_{m}^{\circ }\rangle :1\leq m\leq M\}$ for the LSE is given by*

(4) $$|v_{m}^{\circ }\rangle ={\hat{G}}^{-1/2}|\psi_{m}\rangle ,\;\mathrm{with}\;\hat{G}=\sum_{m=1}^{M}|\psi_{m}\rangle \langle \psi_{m}|.$$

*This basis $\beta^{\circ }$ is known as the square-root measurement [19,20,21,22]. Then, the LSE can be written as*

(5) $$\begin{array}{c} E^{\circ }\left(S\right)=E\left(S,\beta^{\circ }\right)=\frac{1}{M}\sum_{m=1}^{M}{\left(1-\sqrt{\lambda_{m}}\right)}^{2}, \end{array}$$

*where $\lambda_{m}$ is the eigenvalue of the Gram matrix*

(6) $$\begin{array}{c} G=\left[\begin{array}{cccc} \langle \psi_{1}|\psi_{1}\rangle & \langle \psi_{1}|\psi_{2}\rangle & \cdots & \langle \psi_{1}|\psi_{M}\rangle \\ \langle \psi_{2}|\psi_{1}\rangle & \langle \psi_{2}|\psi_{2}\rangle & \cdots & \langle \psi_{2}|\psi_{M}\rangle \\ \vdots & \vdots & \ddots & \vdots \\ \langle \psi_{M}|\psi_{1}\rangle & \langle \psi_{M}|\psi_{2}\rangle & \cdots & \langle \psi_{M}|\psi_{M}\rangle \end{array}\right]. \end{array}$$

## 3. Non-Orthogonality Measure Based on LSE

### 3.1. Maximum and Minimum of LSE

Suppose that *S* consists of orthonormal vectors. Hence, G of *S* is the identity matrix of size *M*. Moreover, the optimal basis $\beta^{\circ }$ is identical to *S*. Therefore, $E^{\circ }\left(S\right)=0$. From definition (1), E(S,β)≥0. Thus, the minimum value of $E^{\circ }\left(S\right)$ is zero.

$E^{\circ }\left(S\right)$ is the solution to the minimization problem of E(S,β) with respect to β for given *S*. However, the maximum of $E^{\circ }\left(S\right)$ for *S* has not been discussed. As mentioned above, the minimum value is attained when *S* consists of orthogonal vectors. Hence, we suppose that the other extreme case, where *S* consists of almost identical vectors, will provide the maximum value. Therefore, we assume that each vector in *S* is close to the barycenter |barycenter〉 for $\beta^{\circ }$. That is,

$$|\psi_{m}\rangle ∼|\mathrm{barycenter}\rangle =\frac{1}{\sqrt{M}}\sum_{\ell =1}^{M}|v_{\ell }^{\circ }\rangle ,$$

and, hence, $|e_{m}\rangle ∼|\mathrm{barycenter}\rangle -|v_{m}^{\circ }\rangle$. This implies

$$E^{\circ }\left(S\right)∼2\left(1-\frac{1}{\sqrt{M}}\right).$$

To give a clear description, we use Equation (5). Applying a simple inequality on the square root ($\sum \sqrt{\cdot }\geq \sqrt{\sum \cdot }$), we have

(7) $$\begin{array}{c} E^{\circ }\left(S\right)=2\left\{1-\frac{1}{M}\sum_{m=1}^{M}\sqrt{\lambda_{m}}\right\}\leq 2\left\{1-\frac{1}{M}\sqrt{\sum_{m=1}^{M}\lambda_{m}}\right\}=2\left(1-\frac{1}{\sqrt{M}}\right). \end{array}$$

Thus, $2(1-1/\sqrt{M})$ is an upper bound of $E^{\circ }\left(S\right)$ for linearly independent *S*.

According to Eldar and Forney [18], the LSE for linearly dependent *S* is given by $E^{\circ }\left(S\right)=2\left[1-\left(1/M\right)\sum_{i=1}^{r}\sqrt{\lambda_{i}}\right],$ where *r* is the rank of G and $\lambda_{i}$ is the nonzero eigenvalue of G. From the convexity of the square root and the inequality used in Equation (7), we have $2\left(1-\sqrt{r/M}\right)\leq E^{\circ }\left(S\right)\leq 2\left(1-1/\sqrt{M}\right)$ for linearly dependent *S*. If all the vectors in *S* are identical, then r=1 and $\lambda_{1}=M$. Therefore, the upper bound $2(1-1/\sqrt{M})$ can be attained by the case that all the vectors in *S* are identical. Thus, the quantity $2(1-1/\sqrt{M})$ can be regarded as the maximum of $E^{\circ }\left(S\right)$ if the identical vector case is allowed. Furthermore, a simple calculation derives the inequality $X_{r}\left(\lambda_{1},\lambda_{2},\lambda_{3},\cdots ,\lambda_{r}\right)\geq X_{r-1}\left(\lambda_{1}+\lambda_{2},\lambda_{3},\cdots ,\lambda_{r}\right)$, where $X_{r}\left(\lambda_{1},\lambda_{2},\lambda_{3},\cdots ,\lambda_{r}\right)=\sum_{i=1}^{r}\sqrt{\lambda_{i}}$ for 2≤r≤M. Therefore, we have

(8) $$2\left(1-\frac{1}{M}X_{r}\left(\lambda_{1},\lambda_{2},\lambda_{3},\cdots ,\lambda_{r}\right)\right)\leq 2\left(1-\frac{1}{M}X_{r-1}\left(\lambda_{1}\;+\;\lambda_{2},\lambda_{3},\cdots ,\lambda_{r}\right)\right).$$

The orthonormal states and the identical state case attain the minimum and maximum values of LSE, respectively. That is, the smallest rank r=1 case gives the maximum, and the full rank r=M case provides the minimum. The inequality above supports this fact. A lower rank has a higher non-orthogonality and vice versa.

### 3.2. A Non-Orthogonality Index of a Collection of Pure State Signals

The range of $E^{\circ }\left(S\right)$ is given by

(9) $$0\leq E^{\circ }\left(S\right)\leq 2\left(1-\frac{1}{\sqrt{M}}\right)\leq 2.$$

Hence, we define the non-orthogonality index (NOI), which is a new measure of the non-orthogonality of a collection of linearly independent pure states, as follows:

(10) $$NOI\left(S\right)\equiv \frac{1}{2\left(1-1/\sqrt{M}\right)}E^{\circ }\left(S\right),$$

where 0≤NOI(S)≤1. The vectors in *S* are almost orthogonal to each other when NOI(S) is approximately equal to 0. Conversely, all vectors in *S* are almost identical when NOI(S) is approximately equal to 1.

## 4. Numerical Simulations

### 4.1. Binary Case

For $S=\{|\psi_{1}\rangle ,|\psi_{2}\rangle \}$,

(11) $$NOI\left(S\right)=\frac{2-\sqrt{1-|\kappa |}-\sqrt{1+|\kappa |}}{2-\sqrt{2}},$$

where the inner product $\kappa =\langle \psi_{1}|\psi_{2}\rangle$. NOI(S)=0 when $|\psi_{1}\rangle$ and $|\psi_{2}\rangle$ are orthogonal (κ=0), and NOI(S)=1 when $|\psi_{1}\rangle =|\psi_{2}\rangle$ (κ=1). From Equation (11), we have

(12) $$\left|\kappa \right|=\frac{1}{2}\left(2-t\right)\sqrt{t(4-t)},\;t=\left(2-\sqrt{2}\right)NOI\left(S\right).$$

**Figure 1:**
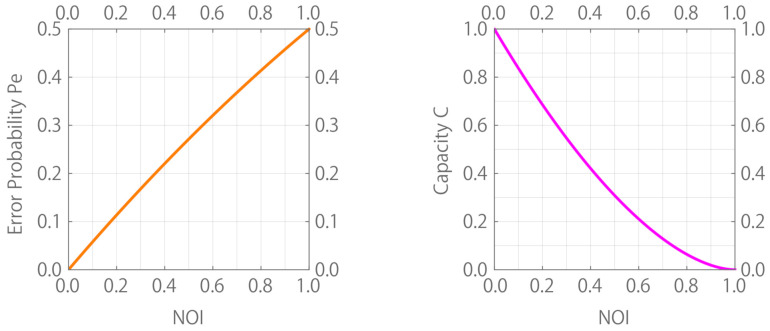
Binary case. (**left**) Minimum error probability $P_{e}$ vs. NOI(S). (**right**) Capacity *C* vs. NOI(S).

The minimum average probability of the quantum detection error is given by $P_{e}=\left(1-\sqrt{1-{\left|\kappa \right|}^{2}}\right)/2$ [23], where we assume that the states are equiprobable. Moreover, the capacity for a binary pure state channel, $b\to |\psi_{b}\rangle$ (b=1,2), is given by $C=-\mu_{+}\log_{2}\mu_{+}-\mu_{-}\log_{2}\mu_{-}$, where $\mu_{\pm }=\left(1\pm |\kappa |\right)/2$ [24]. Figure 1 illustrates the plot of these quantities versus NOI(S) instead of the modulus of the inner product |κ|. The error probability $P_{e}$ is nearly proportional to NOI(S), and the capacity *C* monotonically decreases with respect to NOI(S).

### 4.2. Numerical Simulation I: (Condition-Free)

A simple computer simulation was performed to verify the property 0≤NOI(0)≤1. In this simulation, *M* normalized complex vectors, $|\psi_{m}\rangle =|r_{m}\rangle \in C^{M}$, are randomly generated, and NOI(S) is computed if $S=\{|\psi_{m}\rangle :1\leq m\leq M\}$ is linearly independent. This procedure was repeated 1000 times for each *M*, where M=4,8,16,32,64,128,256. No exceptional values of NOI(S) were observed in this simulation.

### 4.3. Numerical Simulation II: (Almost Orthogonal Case)

A simulation for the case of almost orthogonal quantum states was performed to see how NOI(S) approaches zero.

Let $\beta^{•}=\{|v_{1}^{•}\rangle ,\cdots ,|v_{M}^{•}\rangle \}$ be the standard basis for $C^{M}$. For each *m*, a normalized vector $|r_{m}\rangle \in C^{M}$ is randomly generated and the state vector is set to $|\psi_{m}^{\prime }\rangle =N(|v_{m}^{•}\rangle +\tilde{\delta }|r_{m}\rangle )$, where N is a normalization factor and $\tilde{\delta }$ is a small positive number. When $S^{\prime }=\{|\psi_{1}^{\prime }\rangle ,\cdots ,|\psi_{M}^{\prime }\rangle \}$ is linearly independent, $NOI(S^{\prime })$ and $\delta =\max\{\delta_{1},\cdots ,\delta_{M}\}$ are evaluated, where $\delta_{m}=∥\psi_{m}^{\prime }-v_{m}^{•}∥$. This procedure was repeated 200 times for each $\tilde{\delta }$, where $\tilde{\delta }$ was chosen from 0.001 to 0.3 with step 0.001. Hence, the total number of trials was 60000 for each *M*, where M=8,16,32,64,128,256.

**Figure 2:**
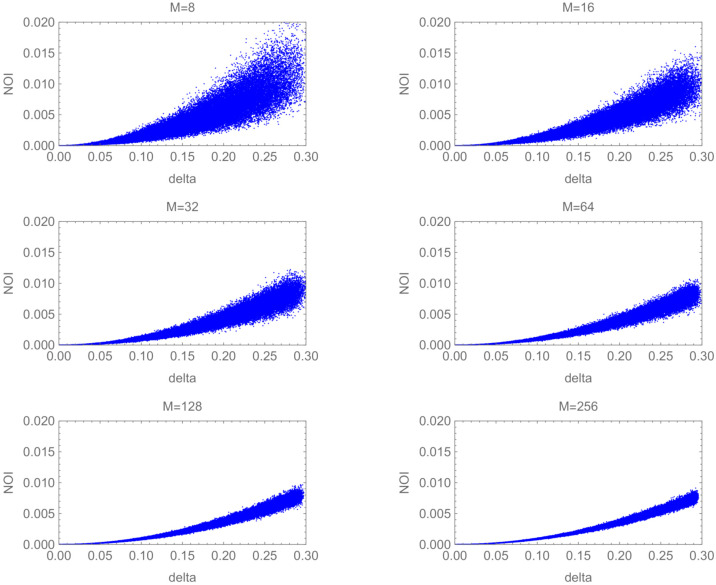
$NOI(S^{\prime })$ vs. δ for almost orthogonal cases.

Figure 2 illustrates the graph of $NOI(S^{\prime })$ versus δ for each *M*. The overall trend of the figures is that $NOI(S^{\prime })$ almost depends on $\delta^{2}$, which reflects the definition of $\delta_{m}$. We observed that the variance of $NOI(S^{\prime })$, which means the dispersion of values at each δ, decreases and the typical value of $NOI(S^{\prime })$ approaches zero when δ approaches zero. Conversely, the smallest value in each δ leaves from the floor line of NOI(S)=0 and the variance of $NOI(S^{\prime })$ increases when δ increases.

Comparing the figures, the variance of $NOI(S^{\prime })$ shrinks as *M* increases. The transition from NOI(S)=0 to NOI(S)=1 in a figure is related to the change in the rank of G. Each graph shows only the case of linearly independent *S*, namely the case of r=M. Taken together with Equation (8), one may infer that the boundary of the plotted points means a borderline of whether the randomly generated vector set is linearly independent or not. Based on this thought, the variance in each δ shows the existing range of linearly independent *S*. Hence, we conjecture that the range of possible values of the NOI for linearly independent sets becomes relatively smaller when *M* increases.

### 4.4. Numerical Simulation III: (Almost Identical Case)

A simulation for the case that the quantum states are almost identical was performed to see how NOI(S) approaches one.

Let $|c\rangle =\left(1/\sqrt{M},\cdots ,1/\sqrt{M}\right)\in C^{M}$. For each *m*, a normalized vector $|r_{m}\rangle \in C^{M}$ is randomly generated and the state vector is set to $|\psi_{m}^{\prime }\rangle =N(|c\rangle +\tilde{\epsilon }|r_{m}\rangle )$, where N is a normalization factor and $\tilde{\epsilon }$ is a small positive number. When $S^{\prime }=\{|\psi_{1}^{\prime }\rangle ,\cdots ,|\psi_{M}^{\prime }\rangle \}$ is linearly independent, $NOI(S^{\prime })$ and $\epsilon =\max\{\epsilon_{1},\cdots ,\epsilon_{M}\}$ are evaluated, where $\epsilon_{m}=∥\psi_{m}^{\prime }-c∥$. This procedure was repeated 200 times for each $\tilde{\epsilon }$, where $\tilde{\epsilon }$ was chosen from 0.001 to 0.3 with step 0.001. Hence, the total number of trials was 60,000 for each *M*, where M=8,16,32,64,128,256.

**Figure 3:**
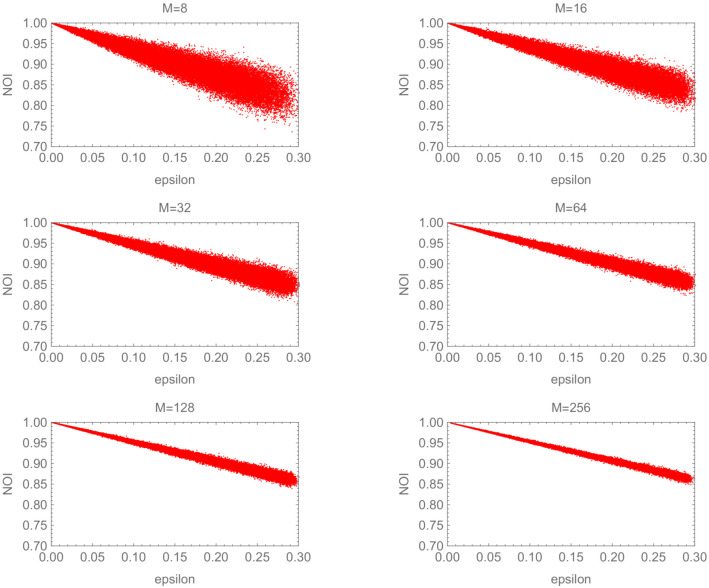
NOI(S) vs. ϵ for almost identical cases.

Figure 3 illustrates the graph of $NOI(S^{\prime })$ versus ϵ. The overall trend of the figures is that $NOI(S^{\prime })$ is linear for ϵ. In each figure, the variance of $NOI(S^{\prime })$ decreases, and the typical value of $NOI(S^{\prime })$ approaches one as ϵ approaches zero. Conversely, the largest value leaves from the ceiling line of NOI(S)=1 and the variance of $NOI(S^{\prime })$ increases when ϵ increases. Comparing the figures, the variance of $NOI(S^{\prime })$ shrinks as *M* increases, as in the almost orthogonal case.

## 5. An Application of the Proposed Technique

Let us consider the case of an *M*-ary PSK coherent state signal as a practical application of the index. As for the *M*-ary PSK coherent state signal, many researchers have studied it in various ways. The performance of the optimal quantum receiver for the PSK signals has been well studied (e.g., [25,26,27,28]). The closed-form expression of the capacity of the pure state channel with the PSK signal was derived in Ref. [29]. The reliability function of the pure state channel with the PSK signal at a high information rate was analyzed in Ref. [30]. Furthermore, an experiment utilizing the $2^{17}$-ary (131072-ary) optical PSK signal was reported in Ref. [15].

An optical signal emitted from a laser can be expressed as a coherent state of light. The coherent state with complex amplitude α [31] is expressed as

(13) $$|\alpha \rangle =\exp\left[-\frac{{\left|\alpha \right|}^{2}}{2}\right]\sum_{n=0}^{\infty }\frac{\alpha^{n}}{\sqrt{n!}}|n\rangle ,$$

where |n〉 is the number state. The average number of signal photons in the state |α〉 is given by $\langle n\rangle ={\left|\alpha \right|}^{2}$. In a communication scenario, the complex amplitude of a coherent state signal is determined based on the signal modulation format. For an *M*-ary PSK coherent state signal, *S* is given by

(14) $$S=\left\{|\alpha_{0}\exp\left[\frac{2m\pi j}{M}\right]\rangle :0\leq m\leq M-1\right\},$$

where $j=\sqrt{-1}$, and the fundamental amplitude $\alpha_{0}$ is assumed to be a positive real number. The *M*-ary PSK coherent state signal is designed to be symmetric on the constellation diagram. Hence, the average number of signal photons does not depend on the probability distribution $p=(p_{0},\cdots ,p_{M-1})$ of the signal. That is,

(15) $$N_{S}=\sum_{m=0}^{M-1}p_{m}{\left|\alpha_{0}\exp\left[\frac{2m\pi j}{M}\right]\right|}^{2}=\alpha_{0}^{2}.$$

In order to compute NOI(S) of the *M*-ary PSK coherent state signal, we use the eigenvalues of G constructed from *S* of Equation (14). In this case, the eigenvalues are given as follows.

(16) $$\lambda_{m}=\sum_{\ell =1}^{M}A_{\left(1,\ell \right)}\cos\left[\Theta_{\left(1,\ell \right)}-\frac{2\pi }{M}m\left(\ell -1\right)\right],$$

where

(17) $$\begin{array}{cc} A_{\left(1,\ell \right)} & =\exp\left[-2|\alpha_{0}{|}^{2}\sin^{2}\left[\frac{\pi }{M}\left(\ell -1\right)\right]\right], \end{array}$$

(18) $$\begin{array}{cc} \Theta_{\left(1,\ell \right)}\; & ={\left|\alpha \right|}^{2}\sin\left[\frac{2\pi }{M}\left(\ell -1\right)\right]. \end{array}$$

**Figure 4:**
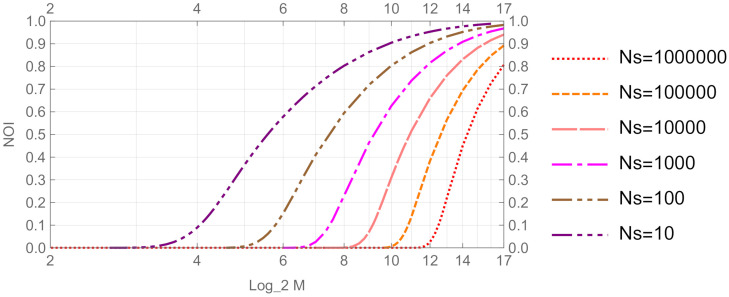
NOI(S) vs. $\log_{2}M$ for *M*-ary PSK coherent state signal.

Figure 4 illustrates the graph of NOI(S) of the *M*-ary PSK coherent state signal versus $\log_{2}M$ (the size of *M* in bits). Typical values of *M* are $2^{4}$ = 16, $2^{6}$ = 64, $2^{8}$ = 256, $2^{10}$ = 1024, $2^{12}$ = 4096, $2^{14}$ = 16,384, $2^{16}$ = 65,536, and $2^{17}$ = 131,072. In this computation, the average number $N_{s}$ of signal photons was between 10 and 1,000,000 photons. From Figure 4, we observe that NOI(S) increases monotonically for *M*. This mutual relationship was observed for all values of $N_{S}$. The non-orthogonality of the states is one of the fundamental properties of a quantum system. Therefore, Figure 4 shows that the *M*-ary PSK coherent state signal exhibits a quantum nature for a significantly large number of signal photons when the total number *M* of the signals is large enough.

**Figure 5:**
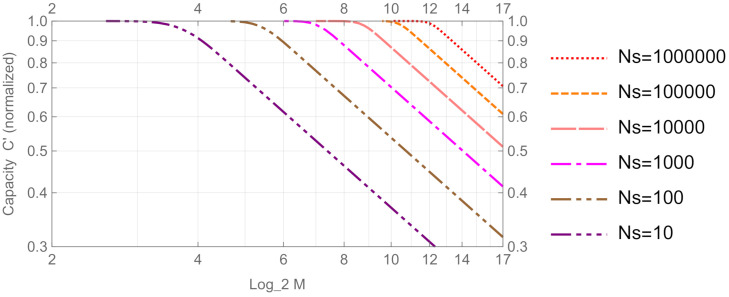
Normalized capacity $C^{\prime }$ vs. $\log_{2}M$ for *M*-ary PSK coherent state signal.

The capacity of the pure state channel $m\to |\psi_{m}\rangle$ for the *M*-ary PSK coherent state signal is analyzed to understand the operational meaning of NOI(S). From Ref. [29], the capacity of this channel is given by

(19) $$C=-\sum_{m=1}^{M}\mu_{m}\log_{2}\mu_{m},\;\mu_{m}=\frac{\lambda_{m}}{M},$$

where $\lambda_{m}$ is given by Equation (16), because the optimal signal distribution to achieve the capacity is a uniform distribution p=(1/M,⋯,1/M). Normalized quantity $C^{\prime }$, which represents the number of Shannon bits per one binary digit of a signal, is obtained by dividing the capacity *C* by $\log_{2}M$. Figure 5 illustrates the graph of the normalized capacity versus $\log_{2}M$. From Figure 4 and Figure 5, we observe that the normalized capacity is maximum (or 1) in the region where NOI(S) is almost zero, and the capacity decreases when NOI(S) increases. Thus, NOI(S) effectively detects the trend of the capacity.

## 6. Conclusions

We have proposed a novel index to measure the non-orthogonality of a collection of linearly independent pure states based on the least squares error criterion in quantum detection theory. We call this index the non-orthogonality index (NOI). First, the non-orthogonality index was analyzed using numerical simulations for binary, condition-free, almost orthogonal, and almost identical cases. The index effectively measured the non-orthogonality of a collection of linearly independent signals from the computer simulations. Next, the non-orthogonality index was applied to the *M*-ary phase-shift keying (PSK) coherent state signal. It was shown that a highly large-scale *M*-ary PSK coherent state signal exhibits high non-orthogonality when the total number of signals is sufficiently large. Furthermore, the index was compared with the capacity of the pure state channel with the PSK signal. Then, we observed that the proposed index effectively detects the trend of the capacity.

In general, a quantum cryptographic system must use a quantum signal set that is unable to distinguish the signals with small detection error or extract much information for an eavesdropper. A simple method is to use single-photon or very weak coherent states. However, this approach has inherent limitations in transmission speed and distance. On the other hand, the coherent state signal having very high power can behave as an almost non-orthogonal signal if the number of signals is sufficiently large. Thus, using a highly large-scale multilevel coherent state signal can create an advantage for legitimate users against the eavesdropper from quantum signal detection. Quantum stream cipher Y00 is a protocol that uses a sufficient number of high-power coherent state signals. Therefore, we conclude that the characterization of a highly large-scale *M*-ary coherent state signal based on the non-orthogonality index provides a basis for understanding cutting-edge optical communication technologies such as quantum stream cipher Y00.

This article discussed the non-orthogonality index in the case of linearly independent pure state signals. Therefore, the generalization of the index remains for future work, which will involve a more precise analysis of linearly dependent cases and the cases of mixed states. In addition, the application to other multilevel coherent state signals such as quadrature amplitude modulation signals will be considered in future work.

## Data Availability

All relevant simulation parameters and equations are within the paper. No experimental data was used in this study.

## Abbreviations

The following abbreviations are used in this manuscript:

LSE	Least squares error
NOI	Non-orthogonality index
PSK	Phase-shift keying
